# A Comprehensive Assessment of Right Ventricular Function in Chronic Thromboembolic Pulmonary Hypertension

**DOI:** 10.3390/jcm12010047

**Published:** 2022-12-21

**Authors:** Stella Marchetta, Tom Verbelen, Guido Claessen, Rozenn Quarck, Marion Delcroix, Laurent Godinas

**Affiliations:** 1 Department of Cardiology, CHC Mont-Légia, 4000 Liège, Belgium; 2 Department of Cardiac Surgery, University Hospitals Leuven, 3000 Leuven, Belgium; 3 Department of Cardiology, University Hospitals Leuven, 3000 Leuven, Belgium; 4 Laboratory of Respiratory Diseases and Thoracic Surgery (BREATHE), Department of Chonic Diseases and Metabolism (CHROMETA), KU Leuven, 3000 Leuven, Belgium; 5 Department of Pneumology, University Hospitals Leuven, 3000 Leuven, Belgium

**Keywords:** chronic thromboembolic pulmonary hypertension, right ventricle, echocardiography, cardiac magnetic resonance imaging

## Abstract

While chronic thromboembolic pulmonary hypertension (CTEPH) results from macroscopic and microscopic obstruction of the pulmonary vascular bed, the function of the right ventricle (RV) and increased RV afterload are the main determinants of its symptoms and prognosis. In this review, we assess RV function in patients diagnosed with CTEPH with a focus on the contributions of RV afterload and dysfunction to the pathogenesis of this disease. We will also discuss changes in RV function and geometry in response to treatment, including medical therapy, pulmonary endarterectomy, and balloon pulmonary angioplasty.

## 1. Introduction

Chronic thromboembolic pulmonary hypertension (CTEPH) is diagnosed in patients presenting with precapillary pulmonary hypertension (PH) with an increased mean pulmonary artery pressure (mPAP) > 20 mmHg at rest, a pulmonary artery wedge pressure (PAWP) ≤ 15 mmHg, and a pulmonary vascular resistance (PVR) > 2 WU [1], together with symptoms and persistence of segmental perfusion defects after three months of effective anticoagulation [2]. Patients with relevant symptoms and segmental perfusion defects in the absence of the aforementioned hemodynamic criteria are diagnosed with chronic thromboembolic pulmonary disease (CTEPD) [2]. CTEPH is classified as PH group 4 in the European Society of Cardiology (ESC)/European Respiratory Society (ERS) guidelines (i.e., PH secondary to pulmonary artery obstruction) [1]. While the incidence of CTEPH varies widely, it is typically diagnosed in 2–3% of patients who have recovered from an acute pulmonary embolism (APE) [3,4].

The persistence of fibro-thrombotic material in the pulmonary vascular bed results in increased PVR and right ventricle (RV) afterload [5]. As the disease progresses, the mPAP increases to maintain blood flow. While the RV initially adapts to the increased afterload, it will eventually become dysfunctional, leading to RV failure [6]. In addition to proximal occlusion of the vascular bed, patients diagnosed with CTEPH frequently develop microvasculopathy [7] due to ongoing shear stress, endothelial dysfunction, and growth of systemic-to-pulmonary anastomoses [5]. Microvasculopathy contributes to the progressive increases in PVR and RV afterload [8].

Current regimens are used to treat CTEPH by targeting the occlusions at various levels [2]. For example, patients with a proximal disease may respond effectively to pulmonary endarterectomy (PEA) which targets and removes occlusions in the main pulmonary arteries (PAs) [9]. By contrast, patients presenting with more distal lesions may benefit from balloon pulmonary angioplasty (BPA) [10]. Microvasculopathy may be targeted with medical therapy, notably with riociguat, which stimulates soluble guanylate cyclase (sGC) [11].

In this manuscript, we will review the pathophysiology of RV dysfunction in CTEPH, with a particular emphasis on points of clinical relevance and the impact of specific medical and surgical treatments.

## 2. Pathophysiology of RV Dysfunction in CTEPH

### 2.1. Pulmonary Vascular Obstruction and Determinants of RV Afterload

**Figure 1:**
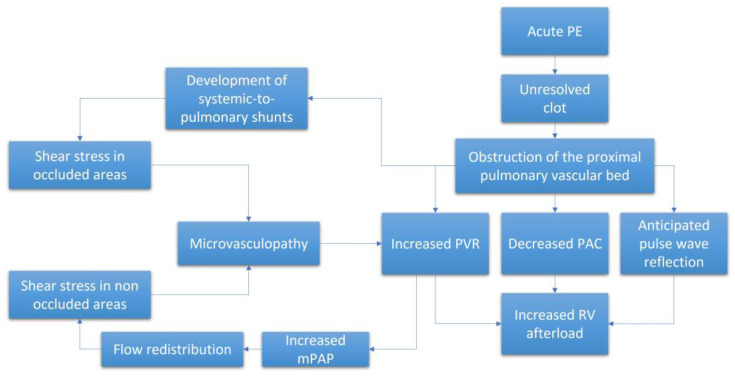
Schematic representation of the pathophysiological process leading to the increased RV afterload. APE = acute pulmonary embolism. mPAP = mean pulmonary artery pressure. PAC = pulmonary artery compliance. PVR = pulmonary vascular resistance. RV = right ventricle.

The pathophysiological mechanisms underlying CTEPH are complex and include both macroscopic and microscopic changes to the pulmonary vascular bed together with pathophysiologic RV responses to the resulting increased afterload (Figure 1). The persistence of fibro-thrombotic material in the lung vasculature increases the PVR and, thus, RV afterload. As the disease progresses, the RV adapts and the mPAP progressively increases to maintain blood flow across the obstructed pulmonary circulation. However, the ability of the RV to adapt will eventually be exceeded. Without effective treatment, the RV will initiate a series of maladaptive responses that will eventually result in right-sided heart failure and death [12]. Of note, the degree of RV afterload that develops is not solely dependent on PVR but also on pulsatile phenomena associated with pulmonary vascular compliance (PVC) and PA impedance (Z) [13] in conjunction with wave reflection and inertance of blood.

Some researchers have suggested that differences in these hemodynamic responses might be used to differentiate between CTEPH, characterized by a proximal macroscopic obstruction and pulmonary arterial hypertension (PAH), in which obstruction is limited to the distal pulmonary vessels with a diameter of less than 1 mm. This point remains controversial. Results from previous studies revealed that an early pulse wave reflection in CTEPH was the result of increased stiffness of the proximal PA [14]. This anticipated wave reflection, which normally reaches the pulmonary valve during diastole, crosses the forward pulse wave at systole; this results in an overall increase in pulse pressure due to the summation of both backward and forward waves. The overall impact of this summation would be decreased blood flow and a net increase in RV workload [14,15,16,17]. However, these results were not entirely confirmed by results from another study, in which a high-fidelity transducer catheter was used to generate pressure measurements in both CTEPH and PAH patients with the same levels of mPAP [18]. While different abnormal pressure-wave reflections were detected when comparing both groups of patients, with results indicating increases in anticipated wave reflection specifically in CTEPH patients, these differences were not sufficient to discriminate between these two diseases. Similarly, results from large animal model studies revealed that pulse pressures and the pulsatile component of RV hydraulic load increased in response to proximal obstruction accompanied by decreases in PVC [19]. However, this model focuses on acute obstruction and thus does not take into account the structural alterations encountered in response to distended proximal circulation that typically results in PH associated with distal obstruction.

Other researchers suggested that patients with CTEPH might exhibit higher PA pulse pressures compared to those diagnosed with PAH and thus potentially associated withreduced PVC [14]. However, these results were not confirmed in other studies in which PA pulse pressures and PVC were reported to be similar in these two conditions [13,18]. These conflicting results might be due to the extent of microvasculopathy in these diseases; distal (as opposed to proximal) remodeling may elicit a more profoundly impaired vascular response to the flow pulsatility [20]. Finally, the relationship between PVR and PVC (the time constant, RC) undergoes little to no variation in patients diagnosed with precapillary PH [21].

### 2.2. Role of Microvasculopathy

Microvasculopathy has been detected in both the occluded and non-occluded lung areas in patients with CTEPH [22]. Various mechanisms have been proposed to explain this phenomenon. Among the possible etiologies, the remodeling of small vessels in non-occluded regions may be the result of increased pressure secondary to the occlusion which will result in more blood flow toward non-occluded lung segments; this will ultimately aggravate the ventilation and perfusion mismatch and trigger shear stress [23,24]. Moreover, the exposure of small vessels to high pressure and the release of vasoactive mediators can lead to endothelial dysfunction and microvasculopathy [8]. Impaired angiogenesis and inflammation have also been involved [25]. Recent studies have highlighted the contributions of systemic-to-pulmonary vascular anastomoses in the induction of microvasculopathy within occluded segments of the lungs [7]. Collectively, these processes contribute to the progressive increases in PVR and RV afterload. The microvascular component may also explain why some CTEPH patients do not respond to surgical treatment (PEA or BPA) and why others can be managed effectively with medical therapy [26]. The information provided in Figure 1 summarizes the pathophysiology underlying the observed increase in RV afterload in patients diagnosed with CTEPH.

### 2.3. RV Adaptation

The RV adapts to progressive increases in afterload via different mechanisms in order to maintain a sufficient cardiac output (CO) at rest and during exercise [27,28]. The adaptative phase is marked by RV remodeling, primarily hypertrophy. This response is consistent with LaPlace’s Law; as applied in this case, RV wall thickness must increase to decrease the wall tension triggered by the RV overload. While undergoing concentric hypertrophy, RV function remains preserved, as well as its capacity to adapt during exercise [29]. Likewise, the stroke volume (SV) is maintained due to the increased contractility conferred by adaptative remodeling. At this point, the right ventricular pulmonary arterial coupling (RV-PAC, assessed as the ratio between end-systolic elastance and arterial elastance, or Ees/Ea) remains constant and adequate to support critical functions [30]. At the cellular level, increased thickness of the RV wall requires both protein synthesis and an increase in cell size secondary to the addition of sarcomeres [31]. At the onset of the disease, these changes are beneficial; RV remodeling and functional adaptability are major determinants of exercise capacity and survival in patients with CTEPH.

### 2.4. RV Failure and the Maladaptive Phenotype

**Figure 2:**
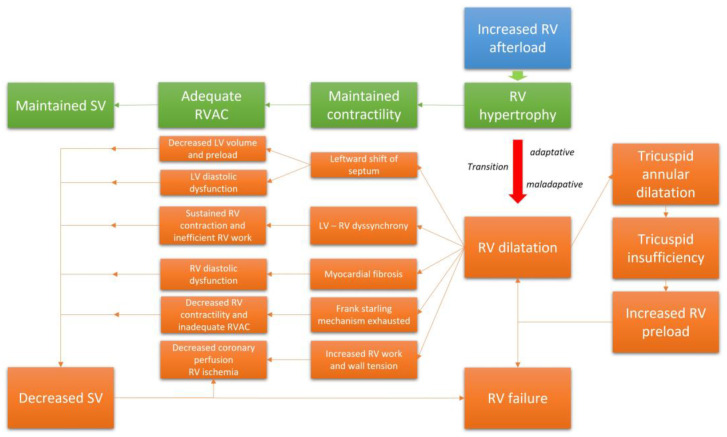
The transition from an adaptative RV phenotype to a maladaptative phenotype.

When the mechanisms leading to adaptation are ultimately exhausted and the RV has no further capacity to adapt, the RV enters into a phase of maladaptive remodeling with volumetric adaptation [32]. This phase is marked by eccentric hypertrophy, RV chamber enlargement, and ultimately dyssynchrony with the left ventricle (LV) [30]. In this phase, the SV is maintained mainly through the Frank–Starling mechanism. The alteration of RV geometry leads to annular dilatation; this will ultimately result in functional tricuspid regurgitation (TR), which will decrease the RVSV and increase the RV preload. Moreover, given the extent of ventricular interdependence within the pericardial space, marked RV dilatation will alter LV geometry due to a leftward shift of the interventricular septum [33]. This displacement will impair LV diastolic function and exacerbate the decrease in CO. The dyssynchrony that develops between the two ventricles will extend the duration of the RV contraction beyond the closure of the pulmonary valves; this will result in additional increased RV wall stress as well as RV work and mechanical inefficiency [34]. This response will also contribute to the leftward shift of the septum, reduced filling space for the LV, and further decreases in the SV [33]. PH may also result in RV diastolic dysfunction due to cardiomyocyte hypertrophy and myocardial fibrosis, which will result in a progressive increase in RV wall stiffness, RV filling pressures, elevated right atrial pulmonary (RAP) pressures, and venous congestion [35,36]. The RV-PAC progressively evolves toward an inadequate relationship characterized by progressive uncoupling [30]. Furthermore, the decrease in systemic blood flow results in deteriorating coronary perfusion despite the increased oxygen demand that develops in response to RV hypertrophy and increased RV work [37]. Increases in RV wall tension and decreases in RV capillary perfusion will both aggravate ischemia. Progressive cardiac dysfunction ensues, with reductions in RV contractility, local ischemia, and myocardial fibrosis that eventually culminate in RV failure [38]. Collectively, this mechanism and relationships explain why RV function is the main leading prognostic indicator in patients with CTEPH. Figure 2 summarizes the evolution of the RV function in CTEPH.

Exercise results in a dramatic increase in RV work because of the need to increase CO to meet the oxygen requirements of active muscles [39]. Therefore, physical activity will create even greater stress and lead to maladaptive RV changes in addition to those already required to maintain adequate SV and CO at rest [40]. This explains why symptoms are mainly encountered during physical activity in the early phase of the disease.

The cellular and tissue responses and mechanisms underlying the transition from compensated RV remodeling to RV failure are complex and not fully understood [41]. Activation of fetal gene expression and angiogenesis mediated by the hypoxia-inducible factor (HIF), and the vascular endothelial growth factor (VEGF) axis, have both been implicated in RV remodeling [42,43,44]; inflammatory and immune-mediated mechanisms have also been described [45,46]. Alterations in myocardial metabolism and activation of the neurohumoral system also contribute to the pathophysiology of the RV maladaptive phenotype [47,48]. Among these changes is a metabolic switch from fatty acid oxidation to anaerobic glycolysis, which is a less efficient mechanism used to provide the ATP required for cardiomyocyte contractions. Similarly, myocardial ischemia coupled with inflammation may lead to collagen deposition and local RV fibrosis [36,49,50]. It is critical to recognize that a hypertrophied RV myocardium has an increased need for oxygen provided by the additional blood supply. However, results from human studies and animal models revealed RV hypertrophy resulted in decreased capillary blood flow [51,52,53] which exacerbated the myocardial ischemia. Collectively, these cellular and molecular abnormalities may trigger mechanisms that lead to fibrosis. In animal models, fibrosis observed in response to maladaptive hypertrophy results from the upregulation of profibrotic growth factors, notably those associated with the TGF-β signaling pathway [54,55]. RV fibrosis has also been detected in cardiac magnetic resonance imaging (cMRI) and histology studies performed in patients with PH [55,56].

While the RV may evolve from an adaptative to a maladaptive phenotype, this change may not be irreversible. A severely dysfunctional RV may improve dramatically in response to significant afterload reduction. This is the underlying rationale for the treatment of CTEPH with PEA, BPA, and potentially lung transplantation. However, it is not clear whether RV function will be fully restored after these treatments, nor do we fully understand what mechanisms might be precluding a complete RV functional repair.

### 2.5. Clinical Relevance of RV Dysfunction in CTEPH

RV dysfunction is an important factor contributing to the symptoms and decreased exercise capacity in patients diagnosed with PH. However, the symptoms due to RV dysfunction that emerge in patients who develop CTEPH after an acute pulmonary embolism (APE) are highly variable and follow a non-linear trajectory. A wide range of PVR and RV dysfunction may be encountered in patients with the same level of pulmonary vascular obstruction and hemodynamic disturbance. Thus, the nature and relevance of the patient symptoms may change greatly, from nearly asymptomatic CTEPD to the most severe and decompensated presentations of CTEPH. This high variation in hemodynamic presentation precludes direct clinical comparison between patients with CTEPH and those with PAH, as the latter present most frequently with high pulmonary pressures and high levels of PVR. 

Several studies have assessed and correlated RV function with symptoms in patients with CTEPH. Among these, persistently increased RV afterload after PEA has been linked to symptoms, including correlations between symptoms and mPAP/CO ratio during exercise [57] and between symptoms and persistence of altered PVC [58]. Abnormal mPAP/CO and RV responses during exercise have also been reported in symptomatic cases of CTEPD [59]. In another study that correlated oxygen consumption (VO_2_) with invasive hemodynamic measurements taken during exercise, the cardiac index (CI) was identified as an independent predictor of impaired VO_2_ and abnormal functional capacity [60]. Similarly, results of a recent study of RV dysfunction based on four echocardiographic RV parameters revealed an association between these outcomes with symptoms and exercise capacity [61]. Taken together, these studies suggest a fundamental role of RV dysfunction as the source of clinical symptoms. In addition to RV dysfunction, the dead-space effect and impaired oxygen extraction in the periphery may provide important contributions to the symptoms of CTEPH [62,63].

RV dysfunction triggered by increased afterload is also the major determinant of prognosis in CTEPH. Results from a large multicenter prospective study of 679 patients revealed that the prognosis of CTEPH patients was positively and independently associated with the New York Heart Association (NYHA) functional class, surgery (i.e., removal of endoluminal obstruction), and RV afterload [64]. In patients who did not undergo surgery, RAP pressure, reflecting the degree of RV failure, was an independent factor associated with disease prognosis [64]. Likewise, results from a series of large retrospective studies revealed that high PVR (both pre-operative and early in the post-operative phase), which is mainly driven by low CO, was associated with poor surgical outcomes, risk of residual PH, and reduced survival [65,66,67,68]. Similarly, results from prospective studies that included 880 CTEPH patients treated with PEA revealed that a low CI and high RAP pressures measured three to six months after PEA were both negative prognosis factors [67]. Other studies reported that CI during exercise and the slope of the mPAP/CO curve were independent predictors of survival in both PAH and CTEPH [60]. Additionally, the pulmonary artery pulsatility index (PAPi), which is the ratio between the pulse pressure and the RAP, has been proposed as a critical parameter linking PVC and RV function. Interestingly, PAPi was inversely correlated with hemodynamic stability; a low PAPi was identified as a negative prognostic factor in patients diagnosed with CTEPH [69]. Collectively, these studies support our current understanding of RV function as a main prognostic marker in CTEPH.

## 3. Evaluation of RV Structure and Function in CTEPH

### 3.1. Right Heart Catheterization (RHC)

RHC remains the “gold standard” for the diagnosis of PH. This method results in accurate measurements of critical pressures, including those measured in the PA (mPAP) and the RA (RAP). Moreover, RHC provides critical information on cardiac function via the direct measurement of CO with either the thermodilution or Fick methods [70]. Assessments of PAP and CO facilitate the calculation of derived measures, including PVR, CI, SV, and PVC. Invasive measurements of the PA flow and RV volumes provide a complex evaluation of pulmonary circulation, including Z and RVAC values, respectively [71,72].

### 3.2. Echocardiography

Echocardiography remains an important tool that can be used to screen patients for CTEPH after APE in cases with persistent symptoms [73]. The systolic pulmonary arterial pressure (sPAP) is commonly estimated from the rate of tricuspid regurgitation (TR) [74]. TR at a velocity > 2.8 m/s corresponds to an sPAP of ~36 mmHg and suggests the possibility of PH [1].

Echocardiography is also in wide use as a means to assess RV function [61]. Signs of adaptative RV function to increased pressure overload can be detected as a thickening of the free wall of the right ventricle (>5 mm), indicating RV hypertrophy [75]. At later stages, maladaptive RV function is revealed by dilatation of the RV (RV diameter >42 mm at the base and >35 mm at the mid-level) together with a flattening of the interventricular septum during systole (and/or in diastole in more severe cases) [76]. Other parameters suggesting PH that can be evaluated by echocardiography include PA acceleration time (PAAT), which can be diagnosed by an RV outflow Doppler acceleration time (<105 ms), followed eventually by the presence of a mid-systolic notch, the latter reflecting an anticipated pulse wave reflection [77]. Signs of severe RV dysfunction include dilatation of the RA (>18 cm^2^) and the inferior vena cava (>21 mm) with decreased inspiratory collapse and hepatic venous reflux [1]. Pericardial effusion may also be detected and is considered to be a sign of very severe disease. RV function can also be assessed based on the tricuspid annular plane systolic excursion (TAPSE, normal value [NV] > 17 mm), tissue Doppler-derived tricuspid lateral annular systolic velocity (RV-S’, NV > 10 cm/s), right ventricular fractional area change (RV-FAC, NV > 35%), and RV myocardial performance index (RV-MPI, NV > 0.4) [75]. Speckle-tracking echocardiography (STE) used to evaluate global longitudinal strain (GLS) of the RV-free wall (RVFW) can provide a highly-sensitive assessment of RV function and local wall contractility [78].

Echocardiography may also be used for a follow-up of CTEPH patients. Results of a recent study revealed the comparatively high sensitivity (68%) and specificity (67%) of transthoracic echocardiography (TTE) for determining the intermediate or high probability of PH and detecting residual PH after PEA [79].

### 3.3. Cardiac Magnetic Resonance Imaging (cMRI)

Cardiac MRI (cMRI) is a useful alternative to echocardiography for the identification and monitoring of patients with PH and to assess RV function [80]. This method provides accurate information regarding RV mass as well as its function and kinetics. In addition to the anatomical information, cMRI also provides a non-invasive assessment of blood flow, including SV, CO, and PA distensibility. However, this method is somewhat less accurate when used to estimate PAP and PVR. Interestingly, structural abnormalities, including RV fibrosis, can be demonstrated and quantified by late enhancement techniques.

## 4. Impact of Pulmonary Thromboendarterectomy on RV Function

### 4.1. RV Structural and Functional Improvement after PEA

PEA is the recommended treatment for patients with CTEPH that is amenable to surgical treatment [1,2]. PEA can be performed to remove an obstruction from the proximal pulmonary vasculature and thus relieve outflow stress on the RV [9,68]. A successful PEA will improve prognosis, symptoms, functional capacity, hemodynamics, and RV function [64,81,82]. Structural and functional RA improvements have been reported early in the history of PEA [83,84], and reverse remodeling is frequently encountered. Comprehensive studies designed to decipher the evolution of RV after PEA have been published [85]. RV volumes, as assessed by echocardiography or cMRI, decrease during the first weeks after the surgery. Invasive hemodynamic measurements also improve with increases in CO and CI and decreases in mPAP, RAP pressures, and PVR. Reductions in PVR (to 70%) and mPAP (to 50%) and normalization of the central venous pressure are frequently detected within the first few days to weeks after PEA. Recovery of RV function occurs somewhat more slowly and frequently remains complete. An early postoperative decrease in RV function is followed by a progressive improvement of both TAPSE and right ventricular ejection fraction (RVEF) during the initial two to three years following the procedure. While the RV wall becomes thinner primarily during the first year, this process continues at a slower pace until three years after the surgery.

Interestingly, the extent of RV reverse remodeling does not depend on preoperative RV function or geometry. Results from a retrospective study that included 17 patients who had undergone PEA revealed a significant improvement in hemodynamics at day 2 [86]. Results from another study revealed similar findings although, in this case, recovery of RV function increased slowly up until six months after PEA and then stopped without achieving full recovery [87]. RV was also evaluated with cMRI and compared to results obtained with healthy controls. At four months after undergoing PEA, patients responded with significant decreases in both RV end-diastolic volume (EDV) and RV end-systolic volume (ESV) with no significant differences observed between treated patients and controls. By contrast, although both RVEFs and RV systolic volumes (SVs) increased, they did not fully normalize. The same was reported for the RV hypertrophy, including a decrease in the thickening of the RVFW that never reached the size of healthy controls. Interestingly, RVEF values correlated significantly with pre-operative disease severity and post-operative improvement, as assessed by total peripheral resistance (TPR) and decreased TPR, respectively. Early decreases in PVR and, therefore, relief of RV overload, is also an independent predictor factor of favorable outcome after PEA [88]. The pulsatile component of the RV, afterload is also improved after PEA in association with an increase in PVC after surgery [88].

TR both before and after PEA has also been examined [89]. Results from a study cohort of 158 patients that underwent PEA revealed that the procedure resulted in a decreased prevalence of TR from 49% to 21% and that residual TR after PEA was independently associated with mortality. Residual TR was also directly associated with RA dilatation, higher RAP pressures, higher estimated sPAP, and significant comorbidities.

PEA not only results in improved RV function and RV volumes, it also alleviates RV septal bowing in left heart chambers, increases LV volumes, and improves LV function. Interventricular dyssynchrony may be normalized, thus further increasing the CO [90]. 

Contractility assessed by GLS and the global circumferential strain were also improved after PEA and correlated with contractility measurements determined by more invasive procedures [91]. Finally, improvement of RV–PA coupling has also been demonstrated one year after PEA [91]. 

Table 1 summarizes the effects of PEA on RV function.

### 4.2. Incomplete Restoration of RV Function

A non-invasive assessment of RV function with TAPSE, RV-S, and RV-MPI documents the incomplete restoration of RV function one year after PEA despite normalization of RV geometry and after a transient depression observed in the early postoperative period [92]. RV wall stress improves after surgery but frequently remains higher than levels detected in healthy subjects. Similar findings were reported in a study focused on RV diastolic function evaluated by echocardiography (i.e., RV E/E’) [92]. Other parameters, including RV mass assessed by cMRI, decrease but do not undergo complete restoration to the pre-disease state. In the early postoperative phase, only a poor correlation was reported between TAPSE and hemodynamic recovery, in contrast to the stronger associations reported for the pre-operative setting. A higher correlation was reported for the RV to LV end-diastolic area ratio. These findings underscore the need for a careful interpretation of the echocardiographic parameters describing RV function in the first several days after PEA that do not predict hemodynamic improvement [102]. This partial restoration of RV function suggests that increased RV afterload may persist and/or an RV plasticity is incomplete, thus precluding its return to a fully restored state. Incomplete improvement in RV function was correlated to exercise limitation [103] and with abnormal hemodynamic responses observed during exercise and an increased mPAP/CO slope despite normalization (or near normalization) of PAP [104].

Incomplete restoration of RV function and RV mass has been linked to evidence of residual PH after PEA [105]. Up to one-third of patients may have persistent (or residual) PH despite an otherwise successful PEA procedure. Persistent PH and poor outcomes can result from incomplete removal of more distal thrombi and/or from concomitant small-vessel disease in patients with the surgically-amenable proximal disease. Changes in PVC after PEA have also been evaluated [39]. In these cases, the absence of improvement has been correlated with poor functional status [103]. Patients presenting with normal hemodynamic parameters at rest after PEA may experience an abnormal increase in PVR and a decrease in PVC during exercise, which have been significantly associated with exercise limitation [57]. Likewise, abnormal resistive and pulsatile pulmonary vascular function after PEA have been associated with a higher mPAP/CO slope [104]; these conditions may respond to the administration of sildenafil. Other parameters of concern include the detection of a systolic notch in the pulmonary artery flow, which suggests the persistence of an early wave flow reflection [106]. This finding has been associated with poor outcomes after PEA [107].

**Figure 3:**
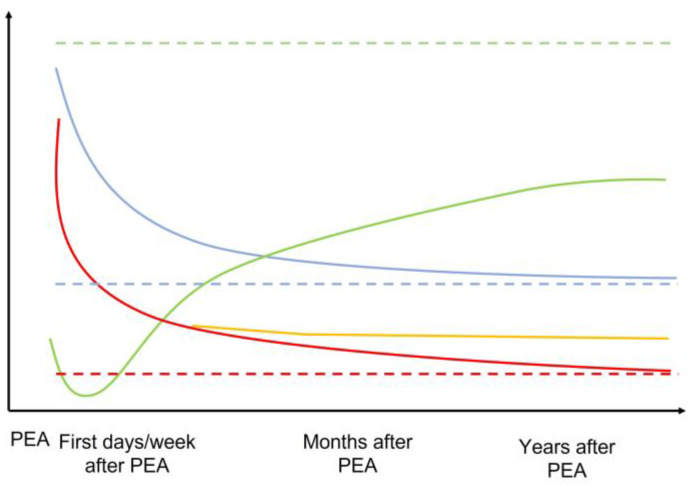
Schematic representation of the evolution of the mPAP, RV geometry, and RV function across time after PEA. The dotted line represents the normal expected values for each parameter. Green = RV function. Blue = RV geometry and dilatation. Red = mPAP if normalized. Orange = mPAP if residual PH after PEA. Dotted lines represent normal situations.

Other explanations that have been proposed to explain the incomplete restoration of RV function after PEA involve the development of myocardial fibrosis and thus intrinsic decreased contractility. Similarly, RVSV also improved after PEA, albeit without a complete restoration to levels exhibited by healthy subjects [108]. Moreover, improvements in these parameters with exercise were also limited and correlated with symptoms. A recent cMRI study of 22 CTEPH patients that was designed to assess regional myocardial function after PEA revealed substantial heterogeneity with respect to recovery of peak systolic longitudinal (i.e., no improvement at 12 days after PEA), radial, and circumferential strain (i.e., improvement at 12 days after PEA) in both ventricles by tissue-tracking analysis despite significant improvements in mPAP [109]. These findings suggest the possibility of differential timing of recovery for different types of myocardial fibers according to their relative overload and may also explain the progressive restoration of RV function after PEA. Figure 3 summarizes the evolution of RV structure and function after PEA.

### 4.3. Effect of Balloon Pulmonary Angioplasty on RV Function

Balloon pulmonary angioplasty (BPA) is an established modality used to treat CTEPH patients [1,2]. BPA is indicated for symptomatic patients who are unable to undergo PEA because of distal disease, persistent/recurrent PH after surgery, or other comorbidities. BPA has been associated with improvements in hemodynamics, exercise capacity, serum levels of N-terminal (NT) pro-brain natriuretic peptide (BNP), and RV function [93,110,111]. Because BPA was developed more recently than PEA, there is only limited evidence available in support of BPA-mediated improvement of right heart function. However, given that both techniques share mechanical aims (i.e., the removal of macroscopic obstruction in the pulmonary vascular bed) [112], the knowledge that has accumulated for PEA may also support the efficacy of BPA in these circumstances.

Results from multiple studies have revealed improvements in hemodynamics together with improvements in both cardiac function and RV afterload via increases in CO and decreases in mPAP and PVR [110,111,113]. Hemodynamic improvements monitored by invasive methods can be translated into improvements in both RV function and geometry [94,95]. In one retrospective study, 30 CTEPH patients who could not undergo surgery were treated with BPA and then followed with cMRI [96]. Improvements were reported in all RV parameters that were assessed by cMRI, except RVSV index. PA flow rate and area were also evaluated in 22 patients; the results of this study revealed increases in average velocity and significant decreases in area, respectively. Similar results were reported from another prospective study, in which 20 patients exhibited decreased RV volumes and improvements in RVEF when evaluated with cMRI [97]. Moreover, the authors reported improvements in interventricular dyssynchrony as assessed by the time to peak of the circumferential strain. Another cMRI study of 29 patients who were evaluated two months after BPA revealed increased pulmonary perfusion, RVEF, RVSV, CO, ventricular mass index, and serum NT-proBNP levels [98]. Another study that included 45 consecutive patients diagnosed with CTEPH revealed significant reverse remodeling of the right heart chambers and improvements in RVFW strain [114]. Interestingly, additional improvements were reported in patients with residual PH after PEA. Speckle tracking parameters, including RVFW longitudinal strain, the RV peak systolic strain dispersion index, and the time to peak longitudinal systolic strain differences also underwent significant improvement after BPA, although they did not reach values reported for healthy subjects [115]. A recent meta-analysis that included 10 studies and 299 patients focused on an evaluation of RV function by cMRI or echocardiography revealed improvements in RV function after BPA, specifically increases in RVEF and decreased RV volumes [116]. While improvements in PVC have not been fully evaluated in patients undergoing BPA, one recent study demonstrated improvements in pulsatile RV afterload of the RV assessed by PVC [113].

### 4.4. Medical Therapy and RV Function

While PEA remains the treatment of choice for CTEPH with a proximal disease, approximately 40% of the patients are considered inoperable secondary to inaccessible vascular obstruction or significant comorbidities [34]. Riociguat, a first-in-class stimulator of sGC, is the only medication that is currently approved for inoperable CTEPH patients or those with persistent/recurrent PH after PEA [11]. Riociguat sensitizes sGC to endogenous nitric oxide (NO) and also directly stimulates sGC independently of NO. Riociguat-mediated activation of sGC results in increased production of cGMP which promotes vasodilation and inhibition of pulmonary vascular remodeling.

Administration of riociguat resulted in improved RV function, reversal of RV hypertrophy, improved vascular remodeling, and decreased myocardial fibrosis in various animal models of PH [117,118,119,120].

Results from a human clinical study revealed that the administration of riociguat results in improved pulmonary hemodynamics associated with improvements in mPAP, CO, CI, and PVR. The changes observed in PVR correlated with increased exercise capacity [121] and remained effective for two years [122]. Interestingly, the administration of riociguat also resulted in decreased systemic vascular resistance in patients diagnosed with CTEPH. This finding explains the hypotension frequently exhibited by patients on this treatment regimen and may contribute to an increase in CO. Moreover, riociguat treatment can reverse the observed enlargement of the right heart chambers (RV and RA), improve TAPSE, RV S’, RV FAC, and RV remodeling as assessed by RV wall thickness through one year after the initiation of therapy [99,123]. RV-GLS was also reduced in CTEPH patients who had undergone treatment with riociguat [100], including those with mild PH after PEA or BPA [101]; these results suggest a mechanism that includes pharmacological recruitment of RV contractile reserve. A recent study of riociguat treatment after BPA demonstrated the beneficial impact of this drug in subjects with mPAP < 30 mmHg. They exhibited improvements in resting PVR and CO and a decrease in the mPAP/CO ratio during exercise [124]. Interestingly, riociguat also had a positive impact on hemodynamics in CTEPH patients that had previously undergone treatment with sildenafil [125]. A recent crossover study that compared BPA and riociguat treatment revealed that BPA was more efficient at decreasing mPAP while riociguat was more effective at improving CO; these results suggested that these modalities target different pathways and elicit different effects [126]. However, no studies have assessed the role of riociguat and its withdrawal after BPA for patients with normalized hemodynamic parameters.

## 5. Conclusions

RV function is an important marker of prognosis in patients diagnosed with CTEPH. The physiopathology of RV dysfunction in this potentially severe disease is complex and involves a multifactorial increase in RV afterload that may be assessed non-invasively by echocardiography and cMRI. Invasive procedures, such as PEA and BPA, may be used to normalize hemodynamics and RV geometry in patients with this disease. However, the observed decrease in afterload does not systematically translate into a normalization of RV function. Persistent RV dysfunction may be linked to ongoing RV overload due to residual obstruction or microvasculopathy. Persistent dysfunction may also be due to diminished levels of intrinsic RV contractility potentially associated with irreversible myocardial fibrosis. Administration of the sGC-activating drug, riociguat, results in improved RV function and may contribute to the ongoing amelioration of RV function after surgery or other interventional treatments.

## Figures and Tables

**Table 1:** Evolution of right ventricular (RV) function after pulmonary endarterectomy (PEA), balloon pulmonary angioplasty (BPA), and riogicuat. cMRI = cardiac magnetic resonance imaging; CO = cardiac output; EDEI = end-diastolic eccentricity index; IVC = inferior vena cava; GLS = global longitudinal strain; RA = right atrium; RVEDAI = right ventricular end-diastolic area index; RVEDV = right ventricular end-diastolic volume; RVSV = right ventricular stroke volume; RVWT = right ventricular wall thickness; TTE = transthoracic echocardiography.

Treatment	Study	Type of RV Evaluation	Parameters	Preoperative	Discharge—1 Month	3–6 Months	12 Months	24 Months
PEA	D’Armini, A.M. et al., 2007 [85]	TTE	FAC TAPSE TR grade II/III IVC EDEI > I	24% 15 mm 78% 22 mm 89%	32% 11 mm 28% 17 mm 14%	33% 14 mm 17% 15 mm 14%	36% 15 mm 23% 14 mm 18%	41% 16 mm 21% 15 mm 21%
		cMRI	RVEDV RVEF RVWT	113 mL 30% 8.4 mm	78 mL 33% 7.8 mm	73 mL 39% 6.9 mm	74 mL 44% 6.3 mm	107 mL 46% 5.8 mm
	Reesink, H.J. et al., 2007 [86]	cMRI	RV-SV RV-EF RV mass	26 mL 34% 49 g/m^2^		36 mL 56% 29 g/m^2^		
	Waziri, F. et al., 2020 [91]	cMRI	RA area RVS RVEDV RV mass RVEF GLS RV CO	14 cm^2^ 65 mL 233 mL 22 g/m^2^ 30% 12.9% 3.9 L/min			8 cm^2^ 71 mL 164 mL 13 g/m^2^ 44% 16.5% 5.1 L/min	
	Surie, S. et al., 2011 [92]	TTE	TAPSE S’ TV annulus CO	19 mm 11.4 cm/s 36 mm 6.1 L/min	12 mm 9.6 cm/s 34 mm 6.2 L/min	15 mm 10 cm/s 34 mm 6 L/min	17 mm 10.3 cm/s 33 mm 6 L/min	
	Iino, M. et al., 2008 [87]	cMRI	RVEF RVSV RVEDV RVESV	31% 57 mL 198 mL 140 ml	47% 61 mL 137 mL 77 ml	52% 66 mL 130 mL 64 ml	52% 67 mL 128 mL 62 ml	
BPA	Fukui, S. et al., 2014 [93]	cMRI	RVSV RVEDV RV mass RVEF	41 mL/m^2^ 130 mL/m^2^ 38 g/m^2^ 34%		37 mL/m^2^ 92 mL/m^2^ 29 g/m^2^ 41%		
	Broch, K. et al., 2016 [94]	TTE	RV basal diameter RVWT FAC RA area TAPSE S’ RV free wall strain	50 mm 6.5 mm 26% 26.5 cm^2^ 19 mm 8.9 cm/s −17%		46 mm 5.6 mm 32% 22.7 cm^2^ 22 mm 10 cm/s −21.7%		
	Tsugu, T. et al., 2015 [95]	TTE	RV basal diameter RVEDV RVSV FAC RVEF TAPSE S’ RV mid free wall strain	33.7 mm 76.4 mL/m^2^ 28.6 mL/m^2^ 22.6% 38% 17.8 mm 11.1 cm/s −19.2%	30.7 mm 64 mL/m^2^ 29.9 mL/m^2^ 32.4% 46.8% 19.2 mm 11.9 cm/s −22.3%			
	Sato, H. et al., 2016 [96]	cMRI	RVSV RVEDV RV mass RVEF	40 mL/m^2^ 104 mL/m^2^ 33.5 g/m^2^ 41%	43 mL/m^2^ 85 mL/m^2^ 26.4 g/m^2^ 51%			
	Yamasaki, Y. et al., 2017 [97]	cMRI	RVSV RVEDV RVEF	38.4 mL/m^2^ 118 mL/m^2^ 35.5%		40.5 mL/m^2^ 33.5 g/m^2^ 42.4%		
	Schoenfeld, C. et al., 2019 [98]	cMRI	RVSV RVEDV RV mass RVEF	43 mL/m^2^ 90 mL/m^2^ 35 g/m^2^ 51%		44 mL/m^2^ 90 mL/m^2^ 36 g/m^2^ 51%		
Riociguat	Marra, A.M. et al., 2015 [99]	TTE	LV eccentricity index RA area TAPSE S’ RVWT IVC	1.2 25 cm^2^ 19.5 mm 11 cm/s 9.5 mm 17 mm		0.95 21 cm^2^ 21.5 mm 12 cm/s 8.3 mm 16.8 mm	1 19.5 cm^2^ 23 mm 13cm/s 8 mm 15.6 mm	
	Murata, M. et al., 2018 [100]	TTE	RV basal diameter RVFAC TAPSE S’ RV GLS IVC	39 mm 35.6 cm^2^ 17.5 mm 10.7 cm/s −13.9% 15 mm		36 mm 39.6 cm^2^ 18.1 mm 11.4 cm/s −17.4% 13.8 mm		
	Murata, M. et al., 2021 [101]	TTE	RV basal diameter RV-EDAI RV FAC TAPSE S’ RV GLS RV dyssynchrony index IVC	39.5 mm 13.5 cm^2^ 33 cm^2^ 18 mm 10.6 cm/s −13.9% 105 ms 9.1 mm			36.5 mm 11.9 cm^2^ 38 cm^2^ 19 mm 11.7 cm/s −17.6% 78 ms 9.8 mm	

## Data Availability

Not applicable.
